# Effectiveness of AI-generated orthodontic treatment plans compared to expert orthodontist recommendations: a cross-sectional pilot study

**DOI:** 10.1590/2177-6709.30.1.e2524186.oar

**Published:** 2025-03-24

**Authors:** Orlando Motohiro TANAKA, Gil Guilherme GASPARELLO, Sergio Luiz MOTA-JÚNIOR, Mohamad Jamal BARK, Jacqueline de Almeida Antunes ROZYSCKI, Rafael Bordin WOLANSKI

**Affiliations:** 1 Center for Advanced Dental Education at Saint Louis University (Saint Louis, USA).; 2 Pontifícia Universidade Católica do Paraná, Medicine and Life Science School (Curitiba/PR, Brazil).; 3 Federal Fluminense University, Department of Orthodontics (Niterói/RJ, Brazil).

**Keywords:** Artificial intelligence, Treatment plan, Diagnosis, Orthodontics, Inteligência artificial, Plano de tratamento, Diagnóstico, Ortodontia

## Abstract

**Introduction:**

Artificial intelligence (AI) has become a prominent focus in orthodontics.

**Objective:**

This study aimed to compare treatment plans generated by AI platforms (ChatGPT, Google Bard, Microsoft Bing) with those formulated by an experienced orthodontist.

**Methods:**

This observational cross-sectional pilot study aims to evaluate the effectiveness of AI-powered platforms in creating orthodontic treatment plans, using a clinical case treated by an experienced orthodontist as a benchmark. A clinical case was selected, and after obtaining informed consent, detailed case information was presented to ChatGPT-3.5, Microsoft Bing Copilot, and Google Bard Gemini for treatment planning. The AI-generated plans, along with the orthodontist’s plan, were evaluated by 34 orthodontists using a questionnaire that included Likert scale and Visual Analog Scale (VAS) items. Statistical analysis was performed to compare the levels of agreement with the proposed treatment plans.

**Results:**

Orthodontists exhibited significantly higher levels of agreement with treatment plans proposed by the orthodontist, compared to those generated by AIs platforms (p < 0.001). Both Likert scale and VAS scores indicated increased confidence in the orthodontist’s expertise in formulating treatment plans. No significant differences were found among the AI platforms, though Google Bard received the lowest mean scores.

**Conclusions:**

Orthodontists demonstrated a higher level of acceptance of treatment plans formulated by human counterparts over those generated by AI platforms. While AI offers significant contributions, the clinical judgment and experience of orthodontists remain essential for thorough and effective treatment planning in orthodontics.

## RESUMO

**Introdução:**

A inteligência artificial (IA) tornou-se um foco proeminente na Ortodontia.

**Objetivo:**

O presente estudo teve como objetivo comparar os planos de tratamento gerados por plataformas de IA (ChatGPT, Google Bard, Microsoft Bing) com o plano formulado por um ortodontista experiente.

**Métodos:**

Este estudo piloto observacional transversal visou avaliar a eficácia de plataformas baseadas em IA na criação de planos de tratamento ortodôntico, usando um caso clínico tratado por um ortodontista experiente como referência. Um caso clínico foi selecionado e, após obter o consentimento informado, informações detalhadas do caso foram apresentadas ao ChatGPT-3.5, Microsoft Bing Copilot e Google Bard Gemini, para planejamento do tratamento. Os planos gerados pela IA, juntamente com o plano do ortodontista, foram avaliados por 34 ortodontistas utilizando um questionário que incluía itens na escala de Likert e na Escala Visual Analógica (EVA). A análise estatística foi realizada para comparar os níveis de concordância com os planos de tratamento propostos.

**Resultados:**

Os ortodontistas apresentaram níveis significativamente mais altos de concordância com o plano de tratamento proposto pelo ortodontista, em comparação com os gerados pelas plataformas de IA (p < 0,001). Tanto as pontuações da escala de Likert quanto da EVA indicaram maior confiança na expertise do ortodontista para formular o plano de tratamento. Não foram encontradas diferenças significativas entre as plataformas de IA, embora o Google Bard tenha recebido as pontuações médias mais baixas.

**Conclusões:**

Os ortodontistas demonstraram um maior nível de aceitação do plano de tratamento formulado pelo ortodontista em comparação aos gerados pelas plataformas de IA. Embora a IA ofereça contribuições significativas, o julgamento clínico e a experiência dos ortodontistas permanecem essenciais para um planejamento de tratamento ortodôntico completo e eficaz.

## INTRODUCTION

Artificial intelligence (AI) has become a focal point of interest, primarily driven by advancements in machine learning techniques that involve intricate layers of artificial neural networks. These networks are trained on extensive datasets, a paradigm commonly referred to as deep learning.^1^ ^,^ ^2^ In Orthodontics, a field search of the PubMed database indicates that since the year 2000, a total of 523 articles have been published on the intersection of “AI and Orthodontics.” Notably, most of these articles (n = 358) emerged between 2021 and 2023, highlighting a pronounced upward trend in AI-related investigations within our field. This mirrors the broader pattern observed in healthcare-related research.^3^

Over recent years, the field of Orthodontics has undergone substantial transformations that have reshaped this specialty.^4^ The shift towards a digital workflow, the advent of temporary anchorage devices, and the emergence of innovative imaging techniques, as well as the use of AI in software’s and hardware collectively contribute to a renewed emphasis on orthodontic care for both patients and professionals.^5^

AI operates trough big data, which typically denotes extensive datasets or the consolidation of data from various sources to identify patterns, enabling the customization of experiences for individuals, as well as enabling machine learning and deep learning. The prominence of big data analytics in the healthcare sector has notably increased in recent years, a trend attributed to the abundance of diverse data sources, advanced computing resources facilitating rapid processing, and a growing emphasis on enhancing the quality of care and clinical outcomes.^6^ ^,^ ^7^

Regarding orthodontics treatment planning, It is crucial to keep in mind that orthodontists might propose different approaches for the same clinical situation. Prior to the start of the treatment process, careful treatment planning must be carried out.^8^ Treatment planning is an intricate process that relies heavily on the orthodontists’ subjective judgment, due to the thorough and deliberate evaluation of numerous variables.^9^ One study have demonstrated that the level of agreement between orthodontists who reviewed identical sets of case records was not very high.^10^

One study has reported the use of AI in diagnosing and creating treatment plans for orthodontics, demonstrating promising results.^11^ A systematic review showed that such automated systems have done remarkably well, achieving accuracy and precision comparable to those of trained examiners.^12^ Another study showed that ChatGPT was effective in delivering high-quality answers related to clear aligners, temporary anchorage devices, and digital imaging within the context of interest of Orthodontics, and may be a valuable auxiliary toll.^13^

Despite the advancements in AI applications within Orthodontics, there remains a gap in the literature regarding the evaluation of AI tools, including ChatGPT (OpenAI, ChatGPT, version 3.5. 2023.), Microsoft Bing Copilot (Microsoft Corporation, Bing Copilot, CA, USA, 2024), and Google Gemini (Bard Experiment, Google Gemini, Mountain View, CA, USA, 2024), in formulating orthodontic treatment plans. Therefore, the objective of this study was to evaluate and analyze the responses generated by these AI platforms for a specific case report, and compare these results with the outcomes from treatments carried out in clinical settings.

## METHODS

This observational cross-sectional pilot study was designed to evaluate the effectiveness of AI-powered platforms in formulating orthodontic treatment plans. It focused on a clinical case treated by an experienced orthodontist with over three decades of experience. After selecting a clinically interceptive case based on specific criteria highlighting typical orthodontic challenges, a informed consent was duly signed by the patient’s legal guardian, in compliance with ethical guidelines. The detailed case description was then uniformly presented to ChatGPT-3.5 (OpenAI, ChatGPT, version 3.5. 2023, accessed February 2, 2024. https://chat.openai.com.), Microsoft Bing Copilot (Microsoft Corporation, Bing Copilot, CA, USA, 2024, accessed February 2, 2024, https://www.bing.com) and Google Gemini (Bard Experiment, Google Gemini, Mountain View, CA, USA, 2024, accessed February 2, 2024. https://bard.google.com) platforms, seeking treatment planning assistance. Subsequently, 34 orthodontists evaluated the proposed treatment plans, including the one formulated by the treating orthodontist, using a standardized framework to ensure a uniform basis for treatment plan generation.

## CASE SELECTION AND DIAGNOSIS

For this study, the following clinical case was selected and described in the AI platforms. 

*“Female patient, 8.10 years old, skeletal Class I. Dental Class I. Overjet (horizontal plane difference) -1.0mm. Overbite (vertical plane relation) null/top to top. Radiographically/Panoramic X-ray, mixed dentition. Maxillary right lateral incisor (12) inclined towards mesial/midline direction. Maxillary right central incisor (11) impacted (not erupted), delayed in its eruption and with mesial/forward inclination. Presence of all successor and supernumerary teeth. Third molar germs in development. Root of tooth 11 fully formed. Cephalometrically/Lateral X-ray. Proportion between facial thirds (mesofacial profile). Slight maxillary retrusion (upper jaw/bone set back). Maxillary and lower incisors slightly protruded (inclined more forward). Clinical/photos/Models: convex facial profile. Tooth 11 still not erupted and maxillary left central incisor (21) crossed (impinging behind the lower incisor). Maxilla slightly constricted. The eruption of the left maxillary lateral incisor is in its initial stages. Possible interference in canines may cause mandibular protrusion.”*

After providing the detailed description, assistance was requested in developing a treatment plan. The responses provided by the AI platforms and by the orthodontist can be seen in the Supplementary Material 1.

## ORTHODONTISTS EVALUATION

To enable a comparative analysis, the treatment suggestions proposed by both the orthodontist and the AI platforms were documented and added in a questionnaire on the Qualtrics digital platform (Salt Lake City, UT, USA), and the following questions were added:

*» “What is your age??”*
*» “What is your gender?”*
*» “For how long have you been practicing orthodontics?* (The responses could be: a) Less than 5 yeas; b) Between 5 and 10 years; c) More than 10 years).

**Figure 1:**
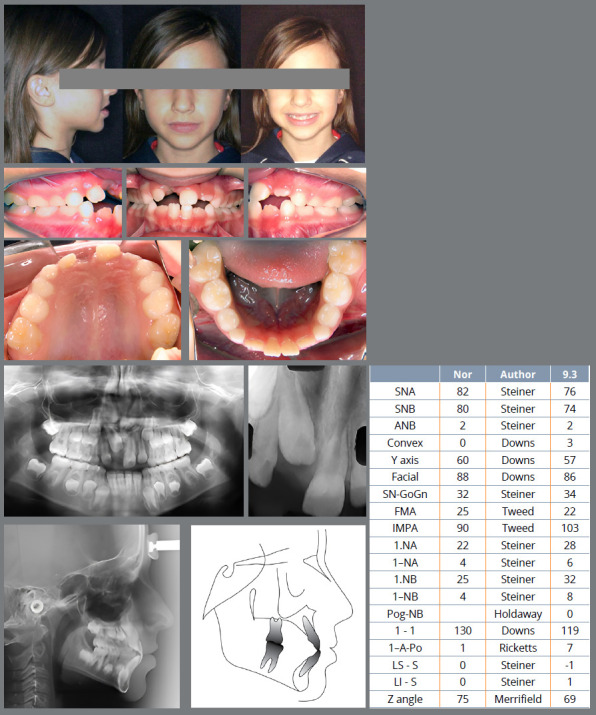
Initial images presented to the orthodontists evaluators.

After those questions, the clinical case initial photographs were displayed (Fig. 1) and then each description from the three AI’s and from the orthodontist were added, with the questions after the description:

*“Regarding to the options, select one related to this treatment plan”.* The answers were presented by means of a 5-point Likert scale (1 = strongly disagree, 2 = disagree, 3 = neutral, 4 = agree, 5 = strongly agree).

*“On a scale of 0 to 100, to what extent do you agree with the above treatment plan?* (0 = completely disagree, 100 = completely agree).

The aim was to assess the evaluators’ unbiased perceptions of each treatment plan’s validity, for this reason, in the description, there was no mention or clues that the texts were generated by AI or by an experience orthodontist.

## STATISTICAL ANALYSIS

The results of the evaluators scores were tabulated in Microsoft Excel software and analyzed in the Statistical Package for Social Sciences v. 25 (SPSS; SPSS Inc., Chicago, IL) program. For the Likert scale, the median, interquartile range (IQR), and full range of scores were determined. ANOVA test was applied to discern differences in scores regarding VAS scores. All statistical analyses were performed with a significance level of p < 0.05, and when conducting Tukey (HSD) and Games-Howell *post-hoc* tests, Bonferroni correction was considered.

## RESULTS

Out of 40 responses collected, 34 were considered valid, as 6 were incomplete due to missing data. Among the validated responses, there were 19 females and 15 males, with a mean age of 40.24 years (range 27-79). From the orthodontists, 14 stated having less than 5 years of experience in Orthodontics, 5 had between 5 and 10 years, and 15 had more than 10 years. Regarding the VAS scores for the question regarding agreement with the treatment plan, significant statistical difference was observed when comparing the plan proposed by an orthodontist to those suggested by ChatGPT (p = 0,001), Google Bard (p < 0,001) and Microsoft Bing (p = 0,001). The mean scores from the orthodontist treatment plan were higher than from those generated by the AI platforms (Table 1). Similarly, for the 5-point Likert scale responses, a consistent pattern was found, with statistical difference in the evaluation of the orthodontist’s plan compared to ChatGPT (p < 0,001), Google Bard (p < 0,001) and Microsoft Bing (p < 0,001). Also, the mean scores for the orthodontist’s treatment plan were higher than those from the AIs (Table 2). No statistical difference was found when comparing only the AIs, although, Google Bard presented lower mean scores compared than ChatGPT and Microsoft Bing.

**Table 1:** VAS scores, ANOVA test and *post-hoc*.

How much do you agree ?				
	Sample size	Mean	Standard deviation	p value
Orthodontist	34	78.38	27.463	p < 0.001
Chat GPT	34	46.94	39.055	
Google Bard	34	40.29	32.059	
Microsoft Bing	34	47.09	30.031	
Tukey HSD				
		Mean difference	Standard error	p value
Orthodontist	Chat GPT	31.441*	7.868	0.001
	Google Bard	38.088*	7.868	0.000
	Microsoft Bing	31.294*	7.868	0.001
Chat GPT	orthodontist	-31.441*	7.868	0.001
	Google Bard	6.647	7.868	0.833
	Microsoft Bing	-0.147	7.868	1.000
Google Bard	orthodontist	-38.088*	7.868	0.000
	Chat GPT	-6.647	7.868	0.833
	Microsoft Bing	-6.794	7.868	0.824
Microsoft Bing	orthodontist	-31.294*	7.868	0.001
	Chat GPT	0.147	7.868	1.000
	Google Bard	6.794	7.868	0.824

**Table 2:** Likert scores, ANOVA test and *post-hoc*.

	Sample size	Mean	Standard error	Median	IQR	Range
Orthodontist	34	4.41	.783	5.00	5.00	2.0 - 5.0
Chat GPT	34	3.09	1.525	3.00	3.00	1.0 - 5.0
Google Bard	34	2.79	1.409	3.00	3.00	1.0 - 5.0
Microsoft Bing	34	3.24	1.350	4.00	4.00	1.0 - 5.0
	Games-Howell					
		Mean difference	Standard error	P value		
Orthodontist	Chat GPT	1.324*	0.294	0.000		
	Google Bard	1.618*	0.277	0.000		
	Microsoft Bing	1.176*	0.268	0.000		
Chat GPT	Orthodontist	-1.324*	0.294	0.000		
	Google Bard	0.294	0.356	0.842		
	Microsoft Bing	-0.147	0.349	0.975		
Google Bard	Orthodontist	-1.618*	0.277	0.000		
	Chat GPT	-0.294	0.356	0.842		
	Microsoft Bing	-0.441	0.335	0.555		
Microsoft Bing	Orthodontist	-1.176*	0.268	0.000		
	Chat GPT	0.147	0.349	0.975		
	Google Bard	0.441	0.335	0.555		

## DISCUSSION

Social media platforms, recognized for their dynamic and interactive nature, are witnessing a surge in the popularity of AI chatbot models. These models are increasingly being utilized by the public as accessible and convenient sources of information for self-care, presenting an alternative avenue for obtaining guidance on various health-related matters. Some studies reported the accuracy of those chatbots for general information about orthodontic treatment, and they look promising.^13^ ^-^ ^15^ Nevertheless, AI is finding applications in Orthodontics that extend beyond cephalometric analysis. The literature on AI’s role in Orthodontics can be categorized into five primary domains: diagnosis and treatment planning, automated landmark detection and cephalometric analysis, evaluation of growth and development, assessment of treatment outcomes, and various other applications.^16^

This pilot study aimed to investigate three different AI chatbots regarding treatment planning, compared to a plan made by an experienced orthodontist. The results showed that the orthodontists agreed much more with the plan made by the orthodontist than those proposed by the chatbots. 

The findings from this study indicate that, despite technological advancements and the increased experience of operators with artificial intelligence (AI) tools for orthodontic planning, there remains a significant preference and greater reliability on treatment plans created by experienced orthodontists. This trend highlights the inherent complexity of orthodontic diagnosis and treatment planning, which often requires a holistic assessment of the patient, including considerations of facial anatomy, growth dynamics, and the interaction between dental and skeletal structures. Although AI chatbots such as ChatGPT, Google Bard, and Microsoft Bing offer an accessible and convenient alternative for gathering information on orthodontic care,^13^ they fail to fully capture the nuance and depth of knowledge required to effectively plan and execute complex orthodontic treatments.

In this study, when comparing only the AI, ChatGPT had slightly higher accuracy than Google Bard, corroborating previous research findings. For example, one study reported that ChatGPT-3.5 outperformed Google Bard in neurosurgery oral board examinations, achieving a higher percentage of accurate responses (62.4% vs 44.2%).^15^ Similarly, another study found that ChatGPT-3.5 was more likely to provide correct or partially correct answers to common lung cancer questions, compared to Google Bard, with approximately a 1.5-fold difference.^17^ Another recent study demonstrated ChatGPT’s effectiveness in improving the readability and simplification of patient information materials, compared to Google Bard: 66.7% of ChatGPT responses were considered safe advice for patients regarding the interpretation of thyroid function test results, compared to 60% of Google Bard responses.^18^

While some studies have highlighted the superior performance of GPT-4 over GPT-3.5^19^ ^,^ ^20^, it is important to note that the present study was limited to freely available public models, excluding GPT-4. This study also included Microsoft Bing AI in the comparison, which presented scores similar to the ChatGPT, whereas Google Bard platform recorded the lowest scores. 

Regarding the options provided by the orthodontist and AI systems, it is well know that rapid maxillary expansion is an effective treatment for improving or correcting maxillary atresia. It has both skeletal and dental effects, allowing for the correction of posterior crossbite, increased arch space, and repositioning of permanent teeth towards the buccal region.^21^ In the treatment plans proposed by the three AI platforms and the experienced orthodontist, only the plans devised by the orthodontist and the Microsoft Bing Copilot made mention of or incorporated or referenced maxillary expansion as part of the therapeutic strategy. However, the proposal from Microsoft Bing Copilot lacked specificity regarding the choice of appliance to address maxillary constriction, a crucial detail for performing the treatment strategy. The inclusion of maxillary expansion in the treatment is considered advantageous for this particular patient, offering potential improvement in maxillary atresia and facilitating space gain within the dental arch. Such interventions are instrumental in rectifying structural limitations and enhancing orthodontic treatment efficacy.

Google Bard specifically suggested the use of a Bionator appliance with springs as a strategic intervention to facilitate the eruption of the impacted tooth. Notably, Bionator appliances are prevalently used within orthodontic practice, due to their efficacy in repositioning the mandible to a more protrusive posture.^22^ However, in this specific patient, the use of this appliance could have negative effects, potentially worsening the patient’s profile, dental alignment, and mandibular positioning. 

On the other hand, the increasing integration of AI in Orthodontics, as demonstrated through its applications in diagnosis and treatment planning, automated landmark detection and cephalometric analysis, and evaluation of growth and development, suggests an untapped potential for these technologies to complement clinical practice. As AI technology advances, its accuracy, reliability, and applicability in specific orthodontic contexts are likely to improve.^16^ ^,^ ^23^

However, it is imperative to recognize AI technology as a auxiliary tool rather then a substitute to the detailed clinical judgment and decision-making skills of experienced orthodontists. Moreover, the education and ongoing updates for professionals regarding the capabilities and limitations of AI are essential to maximize the potential of these tools in enhancing orthodontic care and achieving optimized treatment outcomes. Thus, collaboration between human expertise and artificial intelligence may represent the ideal balance, merging the strengths of both to push boundaries of orthodontic practice forward.

## LIMITATIONS

Since the AI was developed to mimic existing answers, it may fail when encountering atypical patient cases. This represents a fundamental difficulty in the creation of medical AI systems.^24^ The limitations of this pilot study, including its small sample size, lack of control group, and possible selection bias, should be carefully considered when interpreting the results. Despite of these limitations, pilot studies like the present one offer valuable insights, and can contribute to guiding future research on this emerging topic in Orthodontics. 

## CONCLUSIONS

Orthodontists showed higher levels of agreement with the treatment plans proposed by their human counterparts, compared to those generated by AI. Even tough AI can provide valuable insights and suggestions for treatment planning, the expertise and clinical judgment of experienced orthodontists remain crucial in formulating comprehensive and effective treatment plans.

## SUPPLEMENTARY MATERIAL

### SUPPLEMENTARY MATERIAL 1. ANSWERS PROVIDED BY THE AI PLATFORMS AND TREATMENT PLAN MADE BY THE ORTHODONTIST

*“ChatGPT: Based on the provided information, it seems that the patient presents a combination of orthodontic and dental issues that need to be addressed. Here are some suggestions for a treatment plan, but it is crucial for an orthodontist to examine the case in detail, before defining a specific plan: Treatment Objectives: Erupt the upper right central incisor (11). Correct the mesial inclination of the upper right lateral incisor (12). Correct the anterior crossbite with tooth 21. Establish an ideal relationship between upper and lower teeth. Align teeth to correct the negative overjet. Correct the inclination of upper and lower incisors. Correct the slight maxillary retrusion. Treatment Phases: Phase 1: Exposure and Eruption of the Central Incisor (11): Evaluation of the position of tooth 11 and planning for exposure and eruption. Use of orthodontic appliance to create space and allow natural eruption. Phase 2: Correction of the Inclination of the Lateral Incisor (12): Use of fixed orthodontic appliance to correct the mesial inclination of the lateral incisor (12). Phase 3: Anterior Crossbite Correction and Dental Alignment: Correction of anterior crossbite with tooth 21. Use of orthodontic appliance to align teeth and correct overjet. Phase 4: Aesthetic and Functional Adjustments: Fine-tuning to optimize facial aesthetics and occlusal function. Consider possible canine interferences and adjustments to avoid mandibular protrusion. Phase 5: Final Control and Retention: Verification of final occlusion and stability. Prescription of retention appliance to maintain achieved results. Suggested Orthodontic Mechanics: Exposure of Impacted Tooth (11): Use of springs or other devices to create space and allow eruption of the impacted tooth. Fixed Orthodontic Appliance: Use of brackets and wires for alignment and correction of dental inclinations. Overjet and Crossbite Adjustment: Use of intermaxillary elastics and/or other devices to correct negative overjet and anterior crossbite. Retention: Prescription of fixed or removable retention appliances to maintain achieved results.”*

*Google Bard: Detailed Treatment Plan for Patient with Skeletal Class I and Dental Class I: Phase 1: Diagnosis and Planning Medical and Dental History: Gather details about the patient’s medical and dental history, including oral habits, aesthetic and functional concerns, and any history of dental trauma. Investigate for bruxism, mouth breathing, atypical swallowing, parafunctional habits, and family history of malocclusions. Ask about the date of the last dental visit, prophylaxis (cleaning), and oral hygiene habits. Clinical Examination: Assess overall oral health, including the presence of cavities, gum disease, restorations, tooth wear, and malocclusions. Observe the relationship between the jaws and the position of the teeth, including their inclination, crowding, and any gaps (diastema). Analyze the facial profile, the dental midline, and the relationship between the lips and teeth. Palpate the chewing muscles and temporomandibular joint (TMJ), to check for dysfunction. Diagnostic Records: X-rays: Panoramic X-ray to evaluate the complete dentition, including the permanent tooth buds and root development. Lateral cephalometric X-ray (cephalogram) to analyze facial proportions, the jaw relationship, and tooth position relative to the bony base. Periapical X-ray of the impacted central incisor (tooth 11) to assess its position and angulation. Study Models: Impressions of the upper and lower dental arches to create plaster models for detailed analysis of occlusion and tooth crowding. Intraoral and extraoral photographs for case documentation and treatment planning. Diagnosis: Skeletal Class I: ideal anteroposterior relationship between the jaws (Angle’s classification). Dental Class I: neutral occlusion between the teeth (cusp of the upper first molar fits into the groove of the lower first molar). Overjet of -1.0 mm: slight anterior crossbite, with the upper teeth positioned slightly behind the lower teeth. Overbite of 0 mm (edge-to-edge): no vertical overlap between the upper and lower teeth. Upper right lateral incisor (tooth 12) tilted mesially (towards the midline). Upper right central incisor (tooth 11) impacted (unerupted), delayed in eruption, and tilted mesially (forward). All permanent and deciduous teeth present. Third molar buds developing. Root of tooth 11 fully formed. Convex facial profile: increased curvature of the facial profile. Mild maxillary retrusion: maxilla positioned slightly behind the mandible. Upper and lower incisors with slight labial inclination (tipped outwards). Lower incisor overlapping the upper central incisor (tooth 21) in a crossbite. Mild maxillary constriction: narrowing of the upper dental arch. Upper lateral incisors beginning to erupt. Possible canine interference affecting mandibular protrusion. Phase 2: Treatment Planning Objectives: Expose and erupt the impacted upper right central incisor (tooth 11). Correct the inclination of the upper right lateral incisor (tooth 12). Align the teeth and correct the anterior crossbite. Level the curve of Spee (arch curvature). Achieve ideal Class I occlusion, with proper overjet and overbite. Improve facial aesthetics and convex profile. Maintain healthy gums and periodontal tissues. Suggested Mechanics: Exposure and traction of upper right central incisor (tooth 11), using a Bionator appliance, a removable appliance that uses springs to apply gentle and constant force on the impacted tooth, promoting its eruption. Orthodontic traction: after partial eruption of tooth 11, brackets and wires can be used to guide it into its proper position.”*

“Microsoft Bing: *The patient presents a Class I skeletal and dental relationship, with some specific features that require attention in the treatment plan. Let’s consider the available information to develop an appropriate plan: Treatment Objectives: Correct the overjet (horizontal difference between upper and lower teeth). Align the upper and lower incisors. Resolve the crossbite of tooth 21. Accommodate the impacted tooth 11. Improve the facial profile. Suggested Mechanics: Maxillary Expansion - To correct the crossbite of tooth 21 and improve the maxillary relationship. Conventional Fixed Appliance - Use brackets and wires to align the teeth and correct the overjet. Intermaxillary Elastic - Use elastics to bring the impacted tooth 11 into the correct position and adjust the jaw relationship. Reinforced Anchorage - Consider mini-implants or other methods to preserve anchorage during treatment. Radiographic Monitoring: Monitor the development of third molar germs and the eruption of tooth 11. Treatment Sequence: Start with maxillary expansion. Place the fixed appliance and initiate tooth movement. Adjust elastics as needed to correct the overjet and jaw relationship. Evaluate the need for extractions or other additional procedures. Patient Collaboration: Instruct the patient on the importance of following guidelines, using elastics correctly, and attending follow-up appointments.”*

*“Orthodontist: It is suggested, in this first phase of treatment, to perform a slight rapid maxillary expansion with a modified HAAS-type expander (this procedure will be performed provided there is cooperation in the placement and use of the appliance). On the day of the expander appliance fixation, the patient may experience some difficulty in eating and, especially, in swallowing food, but adaptation will gradually occur. The expander appliance should remain in place for 6 to 9 months. The objective of this phase is to slightly increase the perimeter of the upper arch and slightly increase space for the upper right central incisor (11). If necessary, brackets may be bonded to the upper anterior teeth to maintain space for tooth 11. There may be a need for traction of this tooth. In this case, request exposure/opening and bonding of an accessory with a wire to allow support. There is a possibility that one or more teeth may not move, even under the action of controlled orthodontic forces, a situation called ankylosed. In this case, the best clinical approach should be adopted. Reassessment/re-evaluation of the treatment based on radiographs and models should be performed whenever necessary. Extraction of deciduous teeth may be requested if necessary. There is a need for thorough cooperation in oral hygiene and the use of scheduled appliances. Referral to Speech Therapy for evaluation and follow-up, if necessary. At the end of this first phase, after the removal of the expander, all documentation from the initial phase (profile view, panoramic X-ray, upper occlusal view, periapical of the upper incisors, extraoral photos, models) will be requested for evaluation of the results obtained. And also for monitoring the development of successor and supernumerary teeth. As the remaining permanent teeth erupt, one type of appliance or another may be indicated or not to intercept the malocclusion, or to await the opportune time to begin the second phase - corrective treatment (fixed appliance) -, when new complementary exams (X-rays and models) and new planning should be performed.”*
